# Aberrant expression of N-glycolyl GM3 ganglioside is associated with the aggressive biological behavior of human sarcomas

**DOI:** 10.1186/s12885-019-5743-9

**Published:** 2019-06-10

**Authors:** Daniel Pilco-Janeta, Myriam De la Cruz Puebla, Jorge Soriano, Marta Osorio, Iraida Caballero, Adanays Calvo Pérez, Laynes Savon, Natalia Cremades, Rancés Blanco, Adriana Carr

**Affiliations:** 1 Department of Clinical Oncology, “Teodoro Maldonado Carbo” Hospital, 26 de Julio Avenue, 090203 Guayaquil, Ecuador; 2 Sarcoma Translational Research Laboratory, Vall d’Hebron Institute of Oncology (VHIO), 08035 Barcelona, Spain; 3 Department of Clinical Oncology, “Hermanos Ameijeiras” Clinical and Surgical Hospital, San Lázaro Street N° 701 and Belascoaín, 10400 Havana, Cuba; 4 Clinical Trials Unit, National Institute of Oncology and Radiobiology, 29 and F Street Vedado, 10400 Havana, Cuba; 5 Department of Cell Biology and Tissues Banking, National Institute of Oncology and Radiobiology, 29 and F Street Vedado, 10400 Havana, Cuba; 6 Department of Anatomic Pathology, “Hermanos Ameijeiras” Clinical and Surgical Hospital, San Lázaro Street N° 701 and Belascoaín, 10400 Havana, Cuba; 7 Department of Anatomic Pathology, “Teodoro Maldonado Carbo” Hospital, 26 de Julio Avenue, 090203 Guayaquil, Ecuador; 8 Laboratory of Recognition and Biological Activity Assays, 216 Street and 15 Avenue, Atabey, Playa, P.O. Box 16040, 11600 Havana, Cuba; 9 Research and Development Direction, 216 Street and 15 Avenue, Atabey, Playa, P.O. Box 16040, 11600 Havana, Cuba; 10 Atlantic Fellows for Equity in Brain Health, University of California, San Francisco (UCSF), Nelson Rising Lane, Suite 190, San Francisco, CA 94143 USA

**Keywords:** Sarcomas, Immunohistochemistry, N-Glycolyl GM3 ganglioside, Clinicopathological parameters, Overall survival

## Abstract

**Background:**

The aberrant expression of N-glycolyl GM3 ganglioside (NeuGcGM3) in patients with sarcomas was reevaluated by assessing the relation of this molecule with some clinicopathological features and overall survival (OS) of patients.

**Methods:**

Fifty formalin-fixed and paraffin-embedded specimens from patients diagnosed with sarcomas were included. For the evaluation of NeuGcGM3, the 14F7 monoclonal antibody followed by a peroxidase avidin-biotin system was used. Clinicopathological features were obtained from patient records. Survival rates were estimated by the Kaplan-Meier method and compared with the log-rank test. For multivariate analyses, the Cox regression model was used to identify independent prognostic factors for OS.

**Results:**

The majority of samples had high levels of NeuGcGM3 expression (66.0%) that showed statistical correlation with age (*p* = 0.014), TNM stage (*p* = 0.022), histological grade (*p* = 0.013) and proliferation rates (*p* = 0.012). In addition, a tendency for association with tumor depth (*p* = 0.070) was evidenced. In univariate survival analysis, TNM stage (*p* = 0.000), occurrence of metastasis (p = 0.000) and expression of NeuGcGM3 (*p* = 0.034) were significant prognostic factors for OS, while a tendency for association was evidenced for histological grade (*p* = 0.091). Among these variables, only the presence of metastasis (*p* = 0.001) was an independent prognostic factor on multivariate analysis.

**Conclusions:**

The present research suggests the evaluation of NeuGcGM3 expression as a complementary prognostic factor in sarcoma, although our results need to be validated in a larger series and prospective studies. Moreover, our results could support the use of this molecule as a target for immunotherapy.

## Background

Soft Tissue Sarcomas (STS) are a heterogeneous group of tumors that originate from primitive mesenchymal tissue, which account for about 1% of all human malignancies. In spite of advanced gains with multimodality treatments, more than 40% of cases eventually experience tumor recurrence and metastatic spreading [1, 2], which result in a poor overall survival (OS). In this sense, it is of utmost importance to improve our knowledge about the molecular pathogenesis of sarcomas and to develop alternative strategies of treatment against specific molecules. Some of these studies have been focused in total sialic acid content [3] and gangliosides [4].

Gangliosides are sialic acid-containing glycosphingolipids localized in the plasmatic membrane of vertebrate’s cell. In normal human tissues, the N-acetylneuraminic acid (NeuAc) is the most common variant of sialic acid, while the presence of N-glycolylneuraminic acid (NeuGc) is limited due to an inactivating mutation in the cytidine monophosphate-N-acetylneuraminic acid hydroxylase (CMP-NeuAc hydroxylase) gene [5]**.** However, the expression of NeuGc forming part of gangliosides has been found in a variety of human malignancies [6–8], suggesting the potential contribution of these molecules to tumor progression and the metastatic process.

In particular, the aberrant expression of N-glycolyl GM3 ganglioside (NeuGcGM3) was previously reported by immunohistochemistry in a variety of malignant tumors, including pediatric [9] and adult [7, 10] sarcomas. An increased expression of NeuGcGM3 was detected in 59.3–100% of sarcomas, independently of the histological subtype [9], while the presence of this molecule in normal tissues was scarce [7, 11]. This fact permitted to consider NeuGcGM3 as an attractive target for both active and passive immunotherapy. However, the role of NeuGcGM3 in the aggressive biological behavior of sarcomas still remains unclear.

In the present study, it was evaluated for the first time, the association of NeuGcGM3 expression with some clinicopathological features of patients with STS. Moreover, the relation of this molecule with the overall survival of patients was assessed.

## Methods

### Patients and tissue samples

A number of 50 formalin-fixed and paraffin-embedded specimens from patients diagnosed with sarcoma, who underwent tumor surgical resection at the “Hermanos Ameijeiras” General Hospital (Havana, Cuba) between 2006 and 2013, were included. Samples from adult patients of any age, sex, race, histological subtype (except GIST) and any stage of disease were included in the study. Samples from patients who were treated or followed up in other medical institution were excluded.

All cases were staged according to the TNM classification established by the *American Joint Committee on Cancer* (AJCC) [12]. Performance status was evaluated using the ECOG score [13]. Clinical data such as age, gender, tumor size and localization, depth of tumor, the presence of metastasis, disease stage, recurrence, histological subtype and grade of differentiation were obtained from patient records. Overall survival (OS) was measured from the date of surgery to death for any cause or last follow-up and were as calculated for all patients.

This research was conducted after receiving the approved consent by the institutional Ethical Committee.

### Monoclonal antibodies

Sarcomas were immunohistochemically evaluated for a panel including but not limited to vimentin, desmin, pan-actin, S-100 protein, epithelial membrane antigen (EMA), cytokeratins, HNK-1 (CD57), protein gene product (PGP 9.5), CD99, CD34, c-kit, platelet-derived growth factor receptor (PDGFR), CD68 (MIC2), myogenic regulatory protein (MyoD1), h-caldesmon (HCD), alpha**-**1 antitrypsin (A1AT), smooth muscle actin (SMA), muscle specific actin (MSA), myoglobin, myogenin, and Ki-67 (MIB-1). For N-glycolyl GM3 ganglioside, the 14F7 Mab (a highly specific IgG1 against this molecule) produced at the Center of Molecular Immunology (Havana, Cuba) was used [11].

### Immunohistochemical staining

The method previously described [7], was used. Briefly, five-micrometer serial sections from each block were obtained, and the slides were dewaxed in xylene and rehydrated in ethanol following standard procedures. Afterward, the samples were incubated with 14F7 Mab followed by a peroxidase avidin-biotin system. Negative controls were performed substituting primary antibody for washing buffer (TBS). As positive controls, sections of breast adenocarcinoma with known positivity for NeuGcGM3 were used. Enzymatic activity was visualized with a DAB solution and slides were counterstained with Mayer’s Hematoxylin.

### Evaluation of immunostaining

Immunohistochemical results were analyzed for both proportion of stained cells and intensity of 14F7 Mab reactivity. The percentage of positive cells was graded on a scale of 0–3 (0, no staining; 1, 1–50%; 2, 51–75%; and 3, 76–100%). The intensity of reaction was graded on a scale of 0–3; 0, no staining; 1, weak staining; 2, moderate staining; and 3, strong staining. Afterward, an immunoreactive scoring (IRS) was obtained by multiplying the two previously mentioned parameters. Finally, the expression of NeuGcGM3 was divided into low level (IRS < 6) or high level (IRS ≥ 6). For Ki-67 antigen, five areas of greater intensity of staining were subjectively selected and positive and negative cells was counted. Subsequently, the percentage of positive cells (positively stained nuclei/total number of cells × 100) was determined and the measure was grouped as follows: low cell proliferation rate (≤ 10%), moderate cell proliferation rate (11–50%), and high cell proliferation rate (≥ 50%). All slides were assessed by two trained observers (LS, NC) who did not have knowledge of clinical characteristics or outcomes.

### Statistical analysis

The relation between NeuGcGM3 expression and clinicopathological variables were analyzed using the chi-square test. Survival rates were estimated by the Kaplan-Meier method and compared with the log-rank test. For multivariate analyses, the Cox regression model was used to identify independent prognostic factors for OS (overall survival). All statistical analyses were performed with the SPSS program (version 21.0; SPSS Inc., Chicago, USA) and GraphPad Prism 6.0 (GraphPad Software, Inc., San Diego, CA). A *p*-value < 0.05 was considered statistically significant.

## Results

### Patient characteristics

Table 1 shows a summary of clinicopathological features of patients. The median of patient age at presentation was 51 years (ranged from 18 to 82 years). At the time of diagnosis, 19 (38.0%) and 31 (62.0%) of patients presented with 0 and ≥ 1 ECOG performance status, respectively. Seven of the 50 patients (14.0%) showed the presence of metastasis.

**Table 1:** Clinicopathological features of studied patients with sarcomas

Clinicopathological features	No. (%)
Age (years)	
≤ 60	31 (62.0)
> 60	19 (38.0)
Gender	
Women	28 (56.0)
Men	22 (44.0)
Tumor location	
Extremity	33 (66.0)
Trunk	11 (22.0)
Head and Neck	6 (12.0)
Tumor size (cm)	
<5	13 (26.0)
5–10	19 (38.0)
>10	18 (36.0)
Tumor depth	
Superficial	10 (20.0)
Deep	40 (80.0)
TNM Stage	
I	18 (36.0)
II	7 (14.0)
III	18 (36.0)
IV	7 (14.0)
Histological subtype	
Liposarcoma	16 (32.0)
Leiomiosarcoma	8 (16.0)
Pleomorphic/fusocellular	8 (16.0)
Condrosarcoma	4 (8.0)
Extraosseous Ewing sarcoma	4 (8.0)
Other histological subtypes	10 (20.0)
Histological grade (*n* = 47)	
G1	15 (31.9)
G2	4 (8.5)
G3	28 (59.6)
Index of cell proliferation	
≤ 10	20 (40.0)
11–50	21 (42.0)
> 50	9 (18.0)

Legend. *TNM* Tumor node metastasis

Median follow-up for the entire cohort was 2.21 years (range 0.17 to 9.50). After 3-years of follow-up, 22/50 (44.0%) of patients displayed tumor recurrence and 21/50 (42.0%) died within 5 years after diagnosis. According to the first-line therapy used, 37 (74.0%), 9 (18.0%) and 4 (8.0%) of cases received surgery, chemotherapy and supportive care, respectively. The scheme of chemotherapy used as a neoadjuvant and adjuvant regimen were: Ifosfamide/Doxorubicin, Doxorubicin, Scheme P6, Trabectedin, Gemcitabine/Docetaxel and Ifosfamide/Paclitaxel. Adjuvant radiation therapy was delivered in 19 (38%) patients.

### Expression of N-glycolyl GM3 ganglioside

**Fig. 1:**
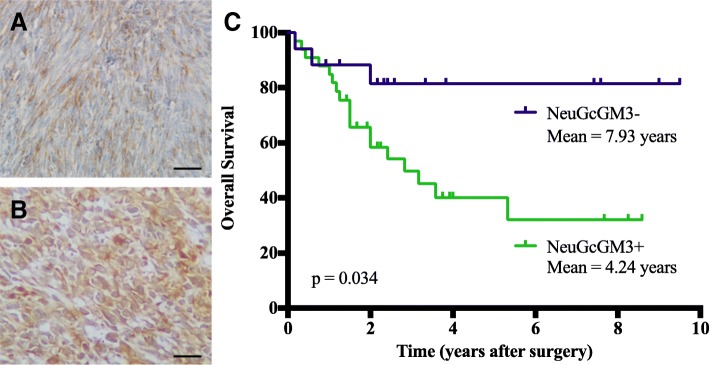
Expression of NeuGcGM3 in human sarcomas. **a** and **b**: Representative photomicrographs of low (IRS < 6) or high (IRS ≥ 6) NeuGcGM3 expression (brown color), respectively. Counterstaining with Mayer Hematoxylin (blue color). Black bar = 100 μm. **c**: Kaplan-Meier survival analyses for overall survival based on NeuGcGM3 expression; NeuGcGM3- (IRS < 6) mean = 7.93 years (navy color); NeuGcGM3+ (IRS ≥ 6), mean = 4.24 years (green color). The immunoreactive score (IRS) was used to generate the dichotomous variable

The expression of NeuGcGM3 was observed in all cases, although a variable intensity and percentage of positive cells was evidenced (Fig. 1a and b). No alterations in the expression pattern of NeuGcGM3 was evidenced when primary treatment options were compared, as previously described by *Blanco* et al.. The staining was detected on both the membrane and cytoplasm of tumor cells with a finely granular staining pattern. The majority of samples had strong intensity (54.0%) and more than 50% of positive cells (92.0%) as shown in Table 2. A strong correlation was found between the percentage of positive tumor cells and staining intensity (Spearman’s correlation coefficient 0.564; *p* < 0.0001). According to the immunoreactive scoring (IRS) 33/50 (66.0%) of cases showed high levels of NeuGcGM3 expression.

**Table 2:** Expression of NeuGcGM3 ganglioside in sarcomas

NeuGcGM3 expression	Number (%)
Intensity of reaction	
Weak	7 (14.0)
Moderate	16 (32.0)
Strong	27 (54.0)
Percentage of positive cells	
≤50	4 (8.0)
51–75	21 (42.0)
≥ 76	25 (50.0)
Immunoreactive score (IRS)	
Low (IRS < 6)	17 (34.0)
High (IRS ≥ 6)	33 (66.0)

Legend. *NeuGcGM3* N-glycolyl GM3 ganglioside, *IRS* Immunoreactive score

### Survival analysis

The results of univariate and multivariate survival analysis are summarized in Table 3. In survival analysis, there was a statistically significant difference in the 5-year OS rates between high and low expression of NeuGcGM3 (45.4% vs. 82.3%; *p* = 0.016), while no significant relation was obtained with Disease-free survival (*p* = 0.346). In addition, univariate analysis showed that TNM stage (*p* = 0.000), occurrence of metastasis (*p* = 0.000), and expression of NeuGcGM3 (*p* = 0.034) (Fig. 1c) were significant prognostic factors for OS, while a tendency for association was evidenced for a histological grade of tumors (*p* = 0.091). Among these variables, only the presence of metastasis (HR = 11.34; 95% CI 2.60–49.4; *p* = 0.001) was an independent prognostic factor on multivariate analysis.

**Table 3:** Univariate and multivariate analysis of overall survival in studied population

Variables	Overall survival		
	Univariate *p* value	Multivariate	
Age	0.695	HR (95% CI)	*p* value
Gender	0.615		
Tumor location	0.306		
Performance status	0.347		
Tumor size	0.305		
Tumor depth	0.118		
Occurrence of metastasis	**0.000**	11.34 (2.60–49.4)	**0.001**
Histological grade	0.091		
Index of cell proliferation	0.757		
TNM stage	**0.000**		
Recurrence	0.309		
NeuGcGM3 expression	**0.034**		

Legend*. HR* Hazard ratio, *CI* Confidence interval, *TNM* Tumor node metastasis, *NeuGcGM3* N-glycolyl GM3 ganglioside. Bold value indicates statistical significance

### Relation of NeuGcGM3 expression with clinicopathological features

**Fig. 2:**
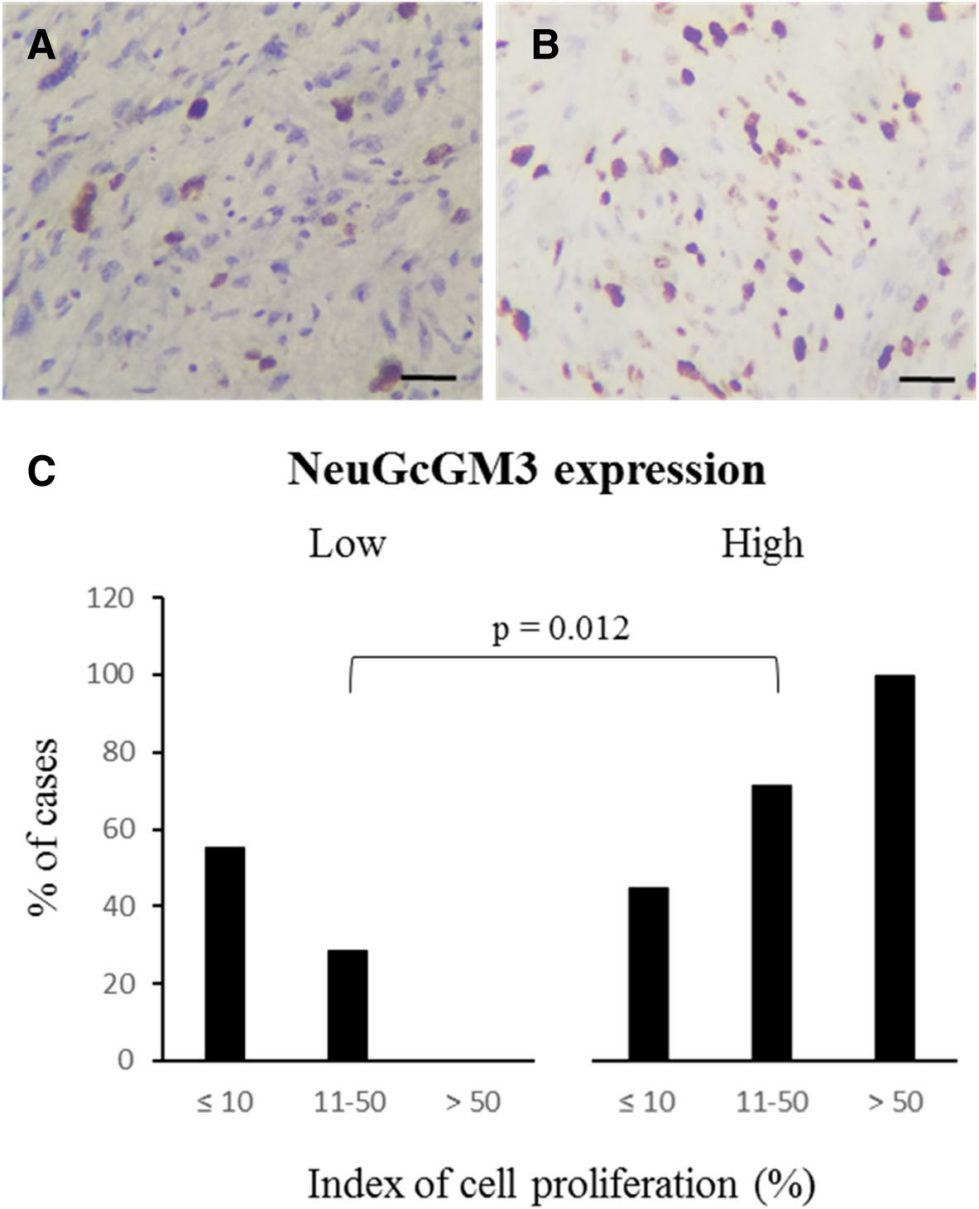
Index of cell proliferation and NeuGcGM3 expression. **a** and **b**: Representative photomicrographs of different levels of nuclear Ki-67 immunostaining (brown color) in sarcomas, as indicative of cell proliferation. Counterstaining with Mayer Hematoxylin (Blue color). Black bar = 100 μm. **c**: Distribution of low (IRS < 6) or high (IRS ≥ 6) NeuGcGM3 expression according to the index of cell proliferation. Cell proliferation rates was grouped as follow: low (≤ 10%), moderate (11–50%) and high (≥ 50%)

The relation of NeuGcGM3 expression with clinicopathological characteristics of patients is shown in Table 4. The level of immunoreactivity (IRS) was associated with age (*p* = 0.014). Interestingly, the presence of this ganglioside was statistically significant increased in advanced clinical stages (*p* = 0.022), high histological grade tumors (*p* = 0.013) as well as in samples displaying an increased index of cell proliferation (*p* = 0.012) (Figs. 2a, b and c). In addition, the expression of NeuGcGM3 was also increased in deeply located tumors when compared with superficial malignancies. However, only a tendency for association was obtained (*p* = 0.070). No significant differences were observed with the rest of the clinicopathological parameters.

**Table 4:** Expression of NeuGcGM3 in relation to selected clinicopathological features

Clinicopathological features	NeuGcGM3 expression (IRS)		*p* value*
	Low	High	
Age (years)			
≤ 60	15	16	0.014
> 60	2	17	
Tumor depth			
Superficial	6	4	0.070
Deep	11	29	
TNM Stage			
I	11	7	0.022
II	1	6	
III	3	15	
IV	2	5	
Histological grade (*n* = 47)			
G1	10	5	0.013
G2	1	3	
G3	6	22	
Index of cell proliferation			
≤ 10	11	9	0.012
11–50	6	15	
> 50	0	9	

Legend. *NeuGcGM3* N-glycolyl GM3 ganglioside; *Chi-square test, *IRS* Immunoreactive score, *TNM* Tumor node metastasis

## Discussion

Although the presence of NeuGcGM3 has been demonstrated in human tumors of epithelial, neuroectodermal, and mesodermal origins [7], the mechanisms that support its expression still remain unclear. The more accepted hypothesis for the presence of NeuGc in human malignancies is associated with its incorporation from exogenous sources to the altered metabolism of malignant cells [14, 15] which is also exacerbate by hypoxia [16]. Along these lines, the expression of NeuGc-containing gangliosides has been shown to be triggered by hypoxic conditions [17]. In particular, *Bousquet* et al. confirmed the capacity of hypoxia to induce NeuGcGM3 in HeLa cells [18].

In the present study, an increased expression of NeuGcGM3 was detected in 66% of human sarcomas, regardless of the histological subtype. The presence of this ganglioside was mainly detected in the surface of malignant cells, although diffusion to cytoplasm was also evidenced, as reported by *Blanco* et al. in these tumors [7]. In previous studies, NeuGc was the predominant sialic acid present on the plasmatic membrane of MG-63 sarcoma cells [19]. Moreover, the expression of cell-associated NeuGc in osteosarcoma cells isolated from patients was demonstrated [20], suggesting the potential role of this molecule in the pathogenesis of osteogenic tumors.

Here, the increased expression of NeuGcGM3 ganglioside was significantly associated with an impaired 5-year overall survival of patients in the univariate analysis. To the best of our knowledge, this is the first report concerning the potential prognostic role of NeuGcGM3 in human sarcomas. However, in the multivariate analysis only the occurrence of metastasis was an independent factor in predicting clinical outcome. Previously, *Lahera* et al. published the association of NeuGcGM3 with the reduced overall survival of patients with colon adenocarcinomas [21], while *Blanco* et al. obtained similar results in NSCLC [22]. In this regard, our results could support the relation of NeuGcGM3 expression with the aggressive biological behavior of sarcomas. Studies regarding the prognostic role of NeuGcGM3 by subtypes of sarcomas with similar clinical behavior are warranted.

The presence of NeuGcGM3 was also related with age of sarcoma patients. In previous research, *Kanduma* et al. reported an increase in the amount of serum total sialic acid in older patients with sarcomas when compared with the rest of groups. Interestingly, these patients also displayed a reduction in the level of natural anti-NeuGc antibodies [3]. Similar to current research, *Lahera* et al. obtained a tendency for association between NeuGcGM3 expression and the age of colon adenocarcinoma patients [21]. Furthermore, *Rodríguez-Zhurbenko* et al. reported a reduction in the production of anti-NeuGcGM3 in older NSCLC patients [23], suggesting the decreased capacity of these patients to effectively fight against tumors. In this sense, the significance of increased expression of NeuGcGM3 in older patients with sarcoma needs further investigation.

In addition, the expression of NeuGcGM3 was significantly increased in high histological grade tumors. In previous studies, *Blanco* et al. reported a relation of NeuGcGM3 expression with an increased histological grade of both malignant gliomas and transitional cell carcinoma of the urinary bladder [22]. It is recognized that high histological grade sarcomas are characterized by an increase in the proliferation rates [24]**,** occurrence of metastasis, and decreased survival rates [25]. Moreover, the presence of NeuGcGM3 was significantly higher in tumors displaying an increased index of cell proliferation. This result aligns with the results obtained by *Blanco* et al. in NSCLC using flow cytometry [22] and could support the preferential expression of NeuGcGM3 in more aggressive forms of sarcomas.

Interestingly, the presence of NeuGcGM3 was increased in deep sarcomas when compared with superficial tumors, although only a tendency for statistical association was obtained. It is known that superficial sarcomas are located exclusively above the superficial investing muscular fascia, while deep tumors are defined as either deep or involving the superficial fascia [26]. Consequently, deep sarcomas are usually characterized by an increased local recurrence and metastatic risk [26, 27] and also with increased tumor size [28], representing a more aggressive form of the disease.

Finally, the presence of NeuGcGM3 was significantly associated with advanced TNM stage, a clinicopathological feature also related with an adverse clinical outcome of patients. Our data is consistent with a previous report of *Lahera* et al. regarding colon adenocarcinomas [21]. It is well known that TNM classification is characterized by increased tumor size and the presence of lymph node and/or distant metastasis. However, no relation of NeuGcGM3 expression with tumor size or occurrence of metastasis separately was obtained, resembling other studies in oral melanoma [29], pediatric retinoblastoma [30], and NSCLC [22]. Although our findings suggest the contribution of NeuGcGM3 to tumor growth and progression, further studies in larger series with better distribution by each stage and histological subtype are required.

## Conclusions

In summary, this study reports for the first time the association of NeuGcGM3 expression with the aggressiveness of human sarcomas. The present research suggests the evaluation of NeuGcGM3 expression as a complementary prognostic factor in sarcoma, although our results need to be validated in a larger series and prospective studies. Moreover, our data could support the use of this molecule as a target for immunotherapy. Interestingly, specific therapies against NeuGcGM3 alone or combined with an anti-EGFR strategy showed promising clinical benefits in sarcoma patients [31]. Lastly, a Phase I clinical trial using the humanized version of 14F7 Mab in Cuban patients with soft-tissue sarcoma has been started.

## Data Availability

The data used to support the findings of this study are available from the corresponding author upon request.

## Abbreviations

AJCC: American Joint Committee on Cancer
ECOG: Eastern Cooperative Oncology Group
EMA: Epithelial membrane antigen
GIST: Gastrointestinal Stromal Tumor
HCD: H-caldesmon
IRS: Immunoreactive scoring
Mab: Monoclonal antibody
MyoD1: Myogenic regulatory protein
NeuAc: N-acetylneuraminic acid
NeuGc: N-glycolylneuraminic acid
NeuGcGM3: N-glycolyl GM3 ganglioside
NSCLC: Non-small-cell lung carcinoma
OS: Overall survival
PDGFR: Platelet-derived growth factor receptor
PGP: Protein gene product
SMA: Smooth muscle actin
STS: Soft Tissue Sarcomas
TBS: Tris-buffered saline
TNM: Tumor, nodule and metastasis

## References

[1] Judson I, Verweij J, Gelderblom H, Hartmann JT, Schöffski P, Blay J-Y (2014). Doxorubicin alone versus intensified doxorubicin plus ifosfamide for first-line treatment of advanced or metastatic soft-tissue sarcoma: a randomised controlled phase 3 trial. Lancet Oncol.

[2] Frezza AM, Stacchiotti S, Gronchi A. Systemic treatment in advanced soft tissue sarcoma: what is standard, what is new. BMC Med [Internet]. 2017;15(1) Available from: http://bmcmedicine.biomedcentral.com/articles/10.1186/s12916-017-0872-y. 10.1186/s12916-017-0872-y PMC5455204 28571564

[3] Kanduma EG, Mukuria JC, Mwanda OW (2007). Serum total sialic acid and Hanganutziu-Deicher antibody in normals and in cancer patients. East Afr Med J.

[4] Roth M, Linkowski M, Tarim J, Piperdi S, Sowers R, Geller D (2014). Ganglioside GD2 as a therapeutic target for antibody-mediated therapy in patients with osteosarcoma: GD2 as a target in osteosarcoma. Cancer..

[5] Irie A, Suzuki A (1998). CMP-N-Acetylneuraminic acid hydroxylase is exclusively inactive in humans. Biochem Biophys Res Commun.

[6] Hayashi N, Chiba H, Kuronuma K, Go S, Hasegawa Y, Takahashi M (2013). Detection of N-glycolyated gangliosides in non-small-cell lung cancer using GMR8 monoclonal antibody. Cancer Sci.

[7] Blanco R, Quintana Y, Blanco D, Cedeño M, Rengifo CE, Frómeta M (2013). Tissue reactivity of the 14F7 Mab raised against N-Glycolyl GM3 ganglioside in tumors of Neuroectodermal, mesodermal, and epithelial origin. J Biomark.

[8] Palomo AG, Santana RB, Perez XE, Santana DB, Gabri MR, Monzon KL (2016). Frequent co-expression of EGFR and NeuGcGM3 ganglioside in cancer: it’s potential therapeutic implications. Clin Exp Metastasis.

[9] Scursoni AM, Galluzzo L, Camarero S, Pozzo N, Gabri MR, de Acosta CM (2010). Detection and characterization of N-Glycolyated gangliosides in Wilms tumor by immunohistochemistry. Pediatr Dev Pathol.

[10] Blanco R. Double Expression of Epidermal Growth Factor Receptor and N-Glycolyl GM3 Ganglioside in Human Malignant Tumors (2017). A Study in Four Different Clinical Scenarios.

[11] Carr A, Mullet A, Mazorra Z, Vázquez AM, Alfonso M, Mesa C (2000). A mouse IgG _1_ monoclonal antibody specific for *N* -Glycolyl GM3 ganglioside recognized breast and melanoma tumors. Hybridoma..

[12] Edge SB, Compton CC (2010). The American joint committee on Cancer: the 7th edition of the AJCC Cancer staging manual and the future of TNM. Ann Surg Oncol.

[13] Oken MM, Creech RH, Tormey DC, Horton J, Davis TE, McFadden ET (1982). Toxicity and response criteria of the eastern cooperative oncology group. Am J Clin Oncol.

[14] Ecsedy JA, Holthaus KA, Yohe HC, Seyfried TN (1999). Expression of mouse sialic acid on gangliosides of a human glioma grown as a xenograft in SCID mice. J Neurochem.

[15] Tangvoranuntakul P, Gagneux P, Diaz S, Bardor M, Varki N, Varki A (2003). Human uptake and incorporation of an immunogenic nonhuman dietary sialic acid. Proc Natl Acad Sci.

[16] Yin J, Hashimoto A, Izawa M, Miyazaki K, Chen G-Y, Takematsu H (2006). Hypoxic culture induces expression of Sialin, a sialic acid transporter, and Cancer-associated gangliosides containing non–human sialic acid on human Cancer cells. Cancer Res.

[17] Alisson-Silva F, Kawanishi K, Varki A (2016). Human risk of diseases associated with red meat intake: analysis of current theories and proposed role for metabolic incorporation of a non-human sialic acid. Mol Asp Med.

[18] Bousquet PA, Sandvik JA, Jeppesen Edin NF, Krengel U (2018). Hypothesis: hypoxia induces de novo synthesis of NeuGc gangliosides in humans through CMAH domain substitute. Biochem Biophys Res Commun.

[19] Tzanakakis GN, Nikitovic D, Katonis P, Kanakis I, Karamanos NK (2007). Expression and distribution ofN-acetyl andN-glycolylneuraminic acids in secreted and cell-associated glycoconjugates by two human osteosarcoma cell lines. Biomed Chromatogr.

[20] Phitak T, Klangjorhor J, Pothacharoen P, Pruksakorn D, Kongtawelert P (2017). Level and distribution of secreted and cell-associated N-acetyl, N-glycolylneuraminic, and deaminoneuraminic acids in osteosarcoma cells isolated from patients. ScienceAsia..

[21] Lahera T, Calvo A, Torres G, Rengifo CE, Quintero S, Arango M del C (2014). Prognostic role of 14F7 Mab immunoreactivity against N-Glycolyl GM3 ganglioside in Colon Cancer. J Oncol.

[22] Blanco R, Domínguez E, Morales O, Blanco D, Martínez D, Rengifo CE (2015). Prognostic significance of N-Glycolyl GM3 ganglioside expression in non-small cell lung carcinoma patients: new evidences. Pathol Res Int.

[23] Rodríguez-Zhurbenko N, Martínez D, Blanco R, Rondón T, Griñán T, Hernández AM (2013). Human antibodies reactive to NeuGcGM3 ganglioside have cytotoxic antitumor properties: clinical immunology. Eur J Immunol.

[24] Samta Shakya SG. Typing and grading of soft tissue tumors and their correlation with proliferative marker Ki-67. J Cytol Histol. 2015;06(03).

[25] Coindre J-M (2006). Grading of soft tissue sarcomas: review and update. Arch Pathol Lab Med.

[26] Kotilingam D, Lev DC, Lazar AJF, Pollock RE (2006). Staging soft tissue sarcoma: evolution and change. CA Cancer J Clin.

[27] Salas S, Stoeckle E, Collin F, Bui B, Terrier P, Guillou L (2009). Superficial soft tissue sarcomas (S-STS): a study of 367 patients from the French sarcoma group (FSG) database. Eur J Cancer.

[28] Rydholm A, Gustafson P. Should tumor depth be included in prognostication of soft tissue sarcoma? BMC Cancer [Internet] 2003 Dec [cited 2019 Jan 12];3(1). Available from: http://bmccancer.biomedcentral.com/articles/10.1186/1471-2407-3-17. 10.1186/1471-2407-3-17 PMC161793 12769830

[29] Zhong Y, Wu Y, Li C, Tang J, Wang X, Ren G (2012). N-Glycolyl GM3 ganglioside immunoexpression in oral mucosal melanomas of Chinese: **t**he 14F7 MAb imunorecognition in oral mucosal melanomas. Oral Dis.

[30] Torbidoni AV, Scursoni A, Camarero S, Segatori V, Gabri M, Alonso D (2015). Immunoreactivity of the 14F7 Mab raised against *N* -Glycolyl GM3 ganglioside in retinoblastoma tumours. Acta Ophthalmol.

[31] Palomo AG, Medinilla AL, Segatori V, Barroso MDC, Blanco R, Gabri MR (2018). Synergistic potentiation of the anti-metastatic effect of anti EGFR mAb by its combination with immunotherapies targeting the ganglioside NGcGM3. Oncotarget..

